# Correlation between body mass index and motor proficiency in Egyptian children: a cross-sectional study

**DOI:** 10.1186/s43161-022-00087-7

**Published:** 2022-06-29

**Authors:** Ahmed S. Awad, Yasser M. Aneis

**Affiliations:** 1 Pediatric Physical Therapy Department, Cairo University, Giza, Egypt; 2 Physical therapy, Basic Sciences Department, Cairo University, Giza, Egypt

**Keywords:** Body mass index, Motor proficiency, Egyptian children, Obesity

## Abstract

**Background:**

Obesity in children is a frequent and serious problem that can impede motor ability performance, necessitating extra attention and early intervention efforts. The purpose of this study was to determine the relation between body mass index (BMI) and motor proficiency in Egyptian children. Two-hundred normal healthy children from both sexes (6 to 8 years old) were enrolled. BMI was defined by dividing weight in kilograms by height in meters squared (kg/m^2^), and motor proficiency was evaluated by Bruininks-Oseretsky Test 2 of Motor Proficiency Short Form (BOT-2 SF). Participants were classified into four categories based on the Centers for Disease Control and Prevention (CDC) cutoff points including underweight, normal, overweight, and obese.

**Results:**

Between-group analysis demonstrated a significant difference between groups where (*χ*^2^ (3) = 131.50, *P* = 0.0001), with the obese group showing the worst motor ability, with mean differences at 95% confidence intervals of 7.44 for underweight, 81.14 for overweight, and 108.92 for obese children. The correlation coefficients of BOT-2 SF and BMI show a significant negative correlation (*R* = −0.723, *P* = 0.0001). Regression analysis revealed that BMI can significantly predict the BOT-2 SF (*F* = 216.94, *P* = 0.0001).

**Conclusion:**

Excess body weight in the period of early childhood in Egyptians has a deleterious effect on motor skill performance; also, children who were normal weight or underweight had higher motor skills than those who were overweight or obese.

## Key messages

- Obesity is a common problem in Egyptian children.
- BMI can predict the level of motor proficiency in children.
- Normal-weight children have superior motor proficiency than overweight or obese children.

## Background

Obesity and lack of activity in children are major issues in today’s society. Sedentary behavior in children has increased sharply, owing primarily to a rise in media consumption [1], and the percentages of people who are overweight or obese have remained stable at a high level [2]. This problem is global, affecting many low- and middle-income countries (LMICs), especially in urban areas [3]. Egypt is one of the LMICs where overweight and obesity among school children are becoming a serious problem, with rates rising from 6 to 15% between 1990 and 2010 [4]. In the USA, Ogden et al. [5] found that overweight and obese children are more likely to be obese as adults and are more likely to develop chronic diseases at an earlier age.

Obese people engage in physical activity at low levels, and inactive people are more liable to become obese; it is a vicious cycle. Whatever the origin or rationale, a large body of evidence suggests that a higher degree of body fat is linked to a lower level of physical performance [6].

Early childhood period is critical for motor skill development [7], as well as establishing a physically active lifestyle [8]. Interaction with the environment is critical for motor development, particularly in infancy. Early in life, environmental inputs, particularly repeated replications of these stimuli, are important clues for a child’s motor memory [9]. Children are more exposed to environmental factors that have negative health consequences; children’s heightened sensitivity to environmental changes is a significant indicator, as it helps us better comprehend present health hazards. Simultaneously, interpreting what is happening to children in the context of environmental causes of disease entails understanding not only the current burden of disease but also future burdens, because many of the effects on children's health are carried over into adulthood, adding to the disease burden [10].

Motor proficiency, a measure of a child’s motor development that can be judged by qualitatively various components of both gross and fine motor skills [11], is one of the most important factors that influences physical activity participation [12]. Obesity in children is related to a decline in motor proficiency, which is a vital competence in this stage of development [13]. Motor proficiency is also found to be related to the status of weight. For example, as compared to normal-weight children, overweight children have large difficulties with core physical abilities and also lower self-concept judgments [14]. The first-grade children’s higher BMI is linked to poor gross motor development. Also, children who engage in more activities in their spare time develop their gross motor skills more effectively [15]. There are, however, a number of additional variables that might affect the motor skills in children, including nutrition, illness, physical activity engagement, and social circumstances [16].

Globally, a few previous reports, with relatively conflicting outcomes, examined the relationships between physical activity, activity loss, motor proficiency, and body mass index [16]. While some demonstrated a negative relationship [14, 17–19], others demonstrated no association at all [20, 21]. These contradictory findings could be attributed to a small sample size or a heterogeneous sample, with most trials using a wide age range.

In Egypt, several studies performed in Cairo [22], Assiut [23], Menoufia [4], Sharkia [24], Sohaj [25], and Port Said [3] discussed the prevalence of childhood overweight and obesity. The prevalence of underweight and overweight in Egyptian school children, as well as their relationship with physical fitness, was also investigated [26]. However, none of the previous studies cleared the impact of both being overweight and being underweight on motor skill proficiency. Therefore, due to the insufficient data and lack of studies in this field in Egypt, it is necessary to conduct this in our research, which will aid in identifying the factors that may obstruct development. So, this will facilitate the creation of developmentally appropriate programs for regulating body weight and achieving optimum movement competence. Consequently, the purpose of the present study was to determine the relationship between BMI and motor proficiency in Egyptian children.

## Methods

This cross-sectional study was performed in 2021 according to the code of ethics of the World Medical Association (Declaration of Helsinki) for experiments involving humans. Ethics approval was obtained from the Faculty of Physical Therapy, Cairo University, Egypt (no.: P.T.REC/012/003400). Initially, 215 Egyptian children were accepted, with 200 healthy children (92 girls and 108 boys) finally participated in the study. They were chosen from elementary schools in North Giza. Fifteen children did not participate because some of their family members were affected by covid-19. Before beginning the study procedures, a formal consent form was completed and received with parental approval for participation in the study and publication of the results. Inclusion criteria were normal children aged from 6 to 8 years, and they can follow directions and comply during evaluation. Excluded children were athletes, children with musculoskeletal or neuromuscular disorders, children with cardiac or respiratory disorders, and children with intellectual disabilities. Participants were classified into four categories for comparison (underweight, normal, overweight and obese).

### Evaluation of BMI

Standard protocols for determining height and weight were followed. The height of children was measured to the closest 0.2 cm without shoes with the use of a handheld stadiometer (SECA, Hamburg, Germany). Children were standing with their heels together, gaze forward, shoulders relaxed, and arms at sides. A calibrated electronic scale was used to measure weight to the closest 0.1 kg (Tanita, Tokyo, Japan). Using the methodology recommended by CDC, BMI (kg/m^2^) was calculated and converted to z-scores [27]. BMI z-scores consider age and gender variances, allowing for comparisons. BMI percentiles in this study were classified into four categories based on age and gender: normal weight < 85th percentile, overweight ≥ 85th percentile, obese ≥ 95th percentile, and underweight (< 5th percentile).

### Evaluation of motor proficiency

Using BOT-2 SF, a well-recognized product-oriented test for assessing gross and fine motor abilities in children aged 4 to 21 years [28]. The BOT-2 SF is a test of motor proficiency that is both valid and reliable [29]. It consists of 14 items drawn from the eight subtests of the entire form (Appendix 1). Seven of the 14 items were accessible in two trials, with the higher trial score being used to calculate the total score. Each child actively completed the 14 items, and after the testing was completed, raw scores were converted into point scores, which were then summed up to obtain the overall point score on the test sheet. To achieve uniformity of scores, eliminate the effect of age, and allow proper comparison of scores, each child’s total point score was converted to a standard score according to the test manual [28].

### Data analysis

The data were checked for normality, variance homogeneity, and the presence of outliers before final analysis. Descriptive analysis utilizing histograms showed that the data violates the parametric assumption as determined by Shapiro-Wilk’s test (*P* > 0.05). The differences between groups in motor proficiency (BOT-2 SF) were examined using the Kruskal-Wallis H test with Dunn’s pairwise post hoc comparison using the Bonferroni correction. Correlations between BOT-2 SF and BMI were further analyzed with Spearman correlation coefficient. A simple linear regression analysis was carried out to create a prediction model for the value of BOT-2 SF in relation to BMI. For all statistical tests, the significance level was established at *P* < .05. All statistical tests were conducted using SPSS for Windows, version 23 (SPSS, Inc., Chicago, IL).

## Results

**Fig. 1:**
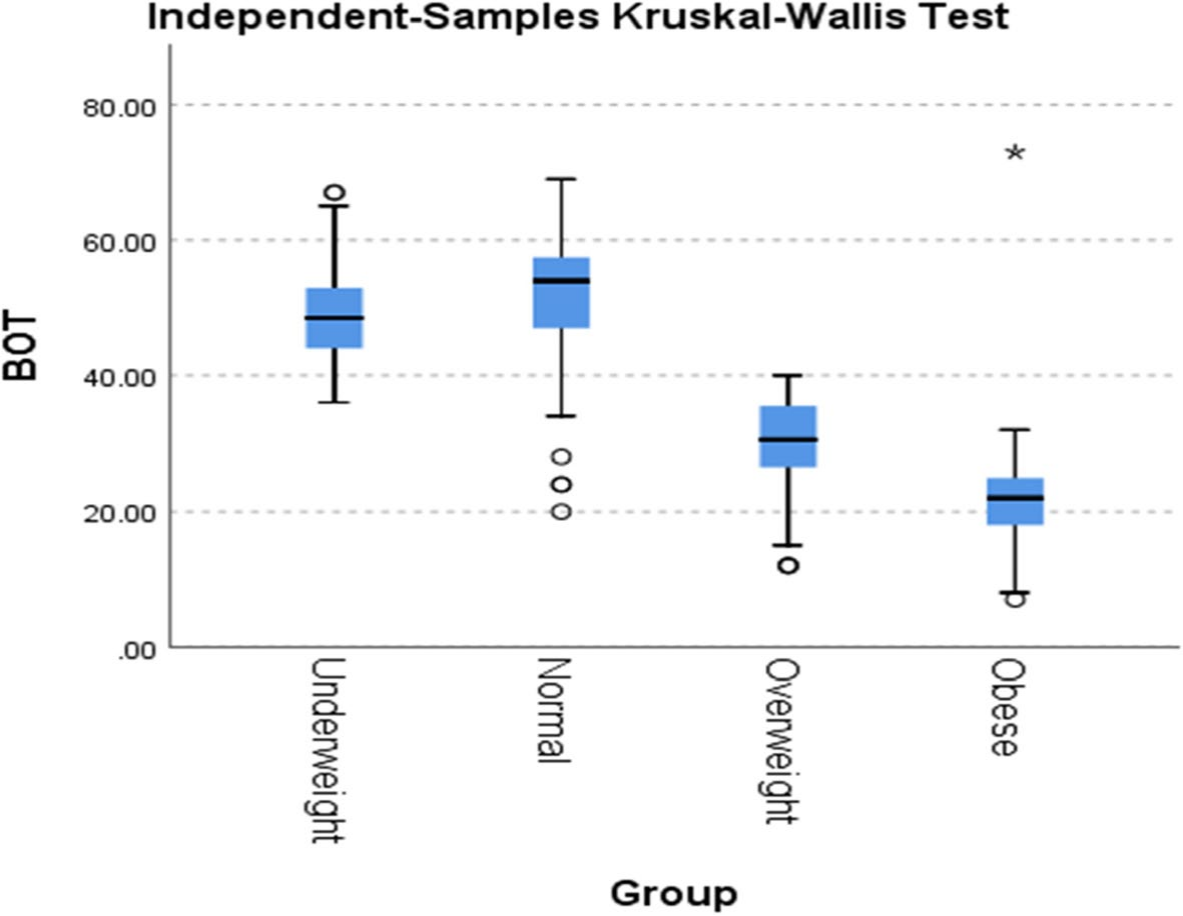
Between-group differences in motor proficiency (BOT-2 SF)

The baseline characteristics and descriptive analysis of all variables addressed in this study are listed in Table 1. There were no statistically significant differences between groups in terms of gender or age (*P* > 0.05). However, regarding BOT-2 SF, there was a considerable difference between groups where (*χ*^2^ (3) = 131.50, *P* = 0.0001). A significant difference between groups in terms of their differential impact on BOT-2 SF was revealed, while normal and underweight children showed better motor proficiency scores than overweight and obese groups. Pairwise comparison analysis revealed a significant difference between groups compared to normal, with the obese group showing the worst motor ability, with mean differences at 95% confidence intervals of (7.44) for underweight, (81.14) for overweight, and (108.92) for obese children (Table 2, Fig. 1).

**Table 1:** Subjects’ demographic and clinical features (mean ± SD)

Group	Number	Age (years)	Gender (Boy)	BMI	BOT-2 SF
**Underweight**	50	6.75 ± 0.78	64%	12.93 ± 0.20	48.98 ± 8.38
**Normal**	52	6.60 ± 0.66	57%	14.94 ± 0.82	50.84 ± 11.05
**Overweight**	48	6.89 ± 0.84	25%	17.73 ± 0.787	28.60 ± 8.73
**Obese**	50	6.68 ± 0.71	66%	19.41 ± 1.19	21.96 ± 9.29
**All cohort**	200	6.73 ± 0.75	53%	16.22 ± 2.63	37.82 ± 15.72

*BMI*, Body mass index, *BOT-2 SF* Motor proficiency

**Table 2:** Between-group differences in motor proficiency (BOT-2 SF)

BOT-2 SF					
**Mean rank**					
**Underweight**	**Normal**	**Overweight**	**Obese**	**Chi-square** *χ*^2^	***p*-value**
141.62	149.07	67.93	40.14	131.50	0.0001
**Pairwise multiple comparison analysis**					
	MD		SE		***p*-value**
Obese-overweight	27.78		11.69		0.017^a^
Obese-underweight	101.48		8.76		0.0001^a^
Obese-normal	108.92		9.50		0.0001^a^
Overweight-underweight	73.69		6.30		0.0001^a^
Overweight-normal	81.14		7.00		0.0001^a^
Underweight-normal	7.44		0.65		0.516

*BOT-2 SF* Motor proficiency; ^a^adjustment for pairwise multiple comparison: Bonferroni

**Fig. 2:**
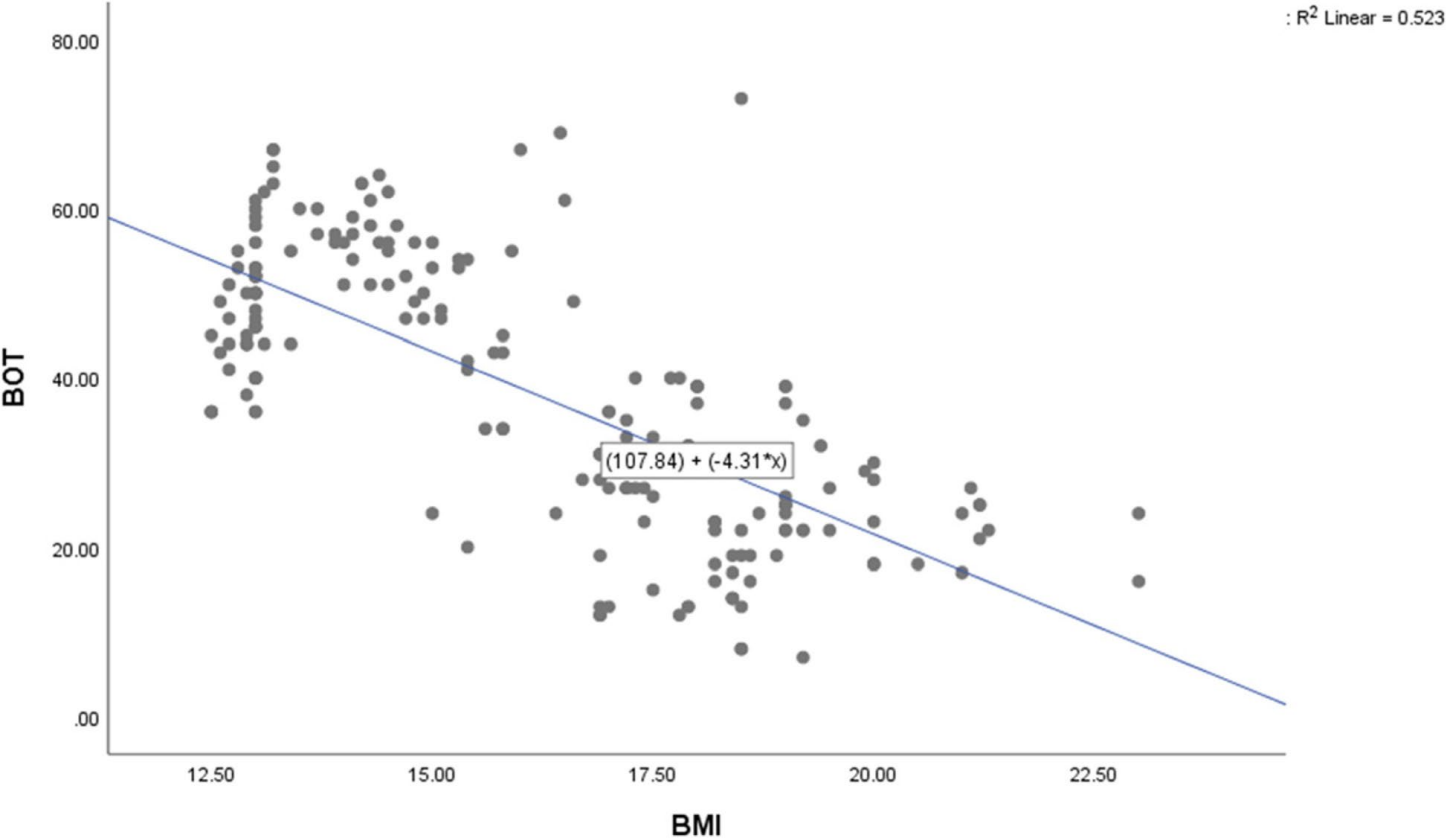
Regression coefficient of BOT-2 SF with predictive model

The correlation coefficients of BOT-2 SF with different degrees of BMI show statistically significant negative correlation (R = 0.723, *P* = 0.0001). Regression analysis revealed that BMI can significantly predict the BOT-2 SF (*F* = 216.94, *P* = 0.0001), (Table 3, Fig. 2). The R2 was 0.520, indicating that the BMI model can account for 52% of the changes in BOT-2 SF. The prediction equation BOT-2 SF = (107.84) + (−4.315×).

**Table 3:** Regression coefficient of BOT-2 SF with predictive model

***R***	***R***^**2**^		B	***t***-value	***p***-value
0.723	0.520	Constant	107.84	22.392	0.0001
		BMI	−4.315	−14.729	0.0001

## Discussion

This study was conducted to determine the relation between BMI and motor proficiency in Egyptian children aged 6–8 years old. Indeed, obesity and overweight in children affect their sports and physical activities involvement and also their capacity to perform motor skills. Our findings showed that BMI and motor proficiency in Egyptian children had a significant negative relationship. This finding is similar to that of Wrotniak et al. [6] who discovered a strong relationship between normalized BMI values and BOT-2 SF scores. Also, Möller et al. [30] discovered significant associations between greater BMI and poorer motor skills. Cheng et al. [31] found that a high relative weight causes future motor skill decreases, not the other way around, confirming our results that obesity has a deleterious effect on motor proficiency in childhood. Hamilton et al. [32] discovered a negative association between preschoolers’ motor skill performance and their BMI scores. This could mean that obesity in preschoolers will hinder and prolong their motor skills in the future. This is reinforced by Stodden et al. [33] who reported that if children are not proficient in performing basic motor abilities (such as running, hopping, single limb support, and so on), they may be insufficiently active at preschool age, which can lead to a rise in their obesity level later on. As a result, obesity and motor skill limitations in this study participants may represent a continuation of their preschool attitude. Moreover, obese children in school before puberty have a greater risk of remaining obese in adulthood and acquiring chronic health problems related to obesity [34]. Obviously, childhood obesity at different ages is a persistent condition that may take a long time to resolve, and this may have an impact on children’s motor skills.

Recent research performed in the Eastern Mediterranean region (EMR) show that the prevalence of overweight among school-aged children (6–18 years old) has reached alarming levels [35], with Kuwait having the highest rate (32%) [36]. There was also an alarming rate of overweight (including obese subjects) among Egyptian children from Assiut, with a prevalence of 24.06% among those aged 6–11 years [26]. Indeed, in most EMR nations, economic improvement has resulted in nutrition transitions in food consumption, centered on energy-dense diets high in fats (i.e., saturated fat, cholesterol, and refined carbohydrates) and low in polyunsaturated fatty acids and dietary fiber. This eating pattern, along with a sedentary lifestyle and elevated levels of stress, has resulted in a significant gain in weight [37]. Previous research has also found a small decline in physical fitness among Egyptian students in primary school age [38].

Our findings also cleared that the Egyptian children who were normal weight or underweight had higher motor proficiency scores than those who were overweight or obese. Logan et al. [20], who discovered that overweight and obese children perform poorly on motor tasks compared to normal and underweight ones, confirm this conclusion. Another study by Gentier et al. [39] said that when compared to their healthy-weight counterparts, overweight and obese children performed badly in manual dexterity, gross, and fine motor skills. According to Abdelkarim et al. [26], the total performance of overweight and obese children was poorer than that of normal-weight children, particularly for balance and motor abilities. Furthermore, these findings are congruent with the findings of Abdel-aziz et al. [40], who found that overweight Egyptian children have considerably lower physical functioning than children with a normal BMI.

Several factors must be considered in order to understand why children with obesity have lower levels of motor proficiency than children of normal weight. Individual, task, and environmental constraints all play a role in motor development, which is a continuing process with multiple variables and interactions [41]. Constrains can affect each other as follows: first, body weight gain changes the body structure and increases the size of various body parts, making actions that are not stationary such as jumping, sprinting, and lifting the weight of one’s own body, more complex and demanding biomechanically [17]. Second, extra body weight has been associated with a drop in perceived physical skill in addition to a reduction in actual physical ability [42]. Obese children’s lower actual and perceived motor skill competence may prevent them from participating in sports and recreational activities that they enjoy with their usual peers [43]. Furthermore, overweight or obese children are more likely to suffer from orthopedic issues, which can result in pain and, as a result, a reduction in motoric function [13]. Stodden et al. [33] stated that the development of motor skill competence encourages regular physical activity, which in turn also promotes motor skill proficiency. These considerations reinforce the current study’s findings, emphasizing the dangers of childhood obesity.

The findings of this study contradict those of Abdelkarim et al. [26], who claimed that, similar to overweight and obese subjects’ lower physical performance, underweight children’s overall performance was also lower than normal-weight children’s, particularly in motor tests based on speed and strength abilities (e.g., sprint, push-ups, and sit-ups). These findings imply that, in the same way that greater focus is paid to overweight and obesity issues, more attention is needed to control abnormal weight loss and prevent its negative impact on children’s physical fitness.

Based on the current study, the percentage of BMI can predict the level of motor performance in early childhood, emphasizing the importance of physical educators in primary schools being aware of children’s weight gains and losses and how to monitor their weight and motor skills to determine the need for referral to specialists who can devise a therapeutic intervention plan. Coordination between specialists and physical educators is critical for facilitating engagement in motor skills that can help children lose weight and maintain an active lifestyle.

### Study limitations

1. Convenience sampling that does not represent the entire population.
2. The sample was taken from a localized population area.

## Conclusions

Motor proficiency in children is adversely associated with BMI, and normal-weight children have superior motor proficiency than overweight or obese children. Early childhood obesity can predict the motor proficiency. Consequently, early intervention programs may aid in the prevention of motor skill decreases; this could have a good impact on children’s physical activity and fitness.

### Recommendations

We recommend future researchers to perform studies on a wider population from different Egyptian environments to draw meaningful conclusion.

## Data Availability

The data collected and/or analyzed during the study are available from the corresponding author on reasonable request and after institutional approval.

## Abbreviations

BMI: Body mass index
BOT-2 SF: Bruininks-Oseretsky Test 2 of Motor Proficiency Short Form
CDC: Centers for Disease Control and Prevention
LMICs: Low- and middle-income countries
EMR: Eastern Mediterranean region
